# Supplementary material for: Bacimethrin, an allelopathic vitamin B1 antagonist, is linked with microbial gene expression patterns in a hypereutrophic watershed
Source: PLoS One. 2025 Nov 21;20(11):e0335861. doi: 10.1371/journal.pone.0335861 (PMC12637900; doi:10.1371/journal.pone.0335861)
Supplement: Attachment — Submitted filename: Review result.docx [file pone.0335861.s019.docx]

In the manuscript “ The allelopathic vitamin B1 antagonist bacimethrin impacts microbial gene expression in a hypereutrophic watershed dominated by cyanobacterial blooms” it is clearly indicated that increased cyanobacterial bloom abundance is associated with increased concentrations of the thiamine and its antagonist, bacimethrin, and increased expression of thiamine biosynthesis genes (*thiC*, *thiF*, and *dxs*). However, some minor errors and obscurities need to be clarified before publishing.

1. The year "2025" in the **Figure 1**B, C caption seems to be a typographical error. Because the Materials and Methods section states that “ samples were collected May 1-3 and August 28-30, 2023, during daylight”.
2. Although linear regressions show that cyanoHAB abundance significantly predicts HMP and bacimethrin concentrations. But the low adjusted R² values (0.18 for HMP and 0.38 for bacimethrin) indicate that there are also other factors controlling the levels of these two chemicals. And authors were unable to identify potential genes responsible for bacimethrin synthesis. However, previous study in M. aeruginosa has suggested that genes encoding glycosyltransferase, methyltransferase, and thiaminase I may be involved in bacimethrin production, like their homologs in the bcm gene cluster found in Clostridium botulinum.
3. Why did you set a threshold of >1,000 cells mL⁻¹ for CyanoHAB high and low?
